# Diagnostic Utility and Risk of Malignancy Stratification Using the World Health Organization (WHO) Reporting System for Lung Cytopathology: A One-Year Institutional Study

**DOI:** 10.7759/cureus.96194

**Published:** 2025-11-06

**Authors:** Amulya Boddapati, Harika Mandava, Inuganti Venkata Renuka, Sudhakar Ramamoorthy, Leela Lahari Arikathota

**Affiliations:** 1 Pathology, NRI Medical College, Guntur, IND; 2 Pathology, Mamata Medical College, Khammam, IND

**Keywords:** bronchoalveolar lavage (bal), diagnostic categories, endobronchial ultrasound-guided transbronchial needle aspiration (ebus-tbna), fine-needle aspiration, international academy of cytology, lung cytology, risk of malignancy, suspicious for malignancy, who reporting system for lung cytopathology

## Abstract

Introduction

The World Health Organization (WHO) recently introduced a standardized Reporting System for Lung Cytopathology, classifying respiratory cytology into five diagnostic categories with corresponding risk of malignancy (ROM) and management guidelines. This study applies the WHO system to categorize respiratory tract cytology specimens and assess ROM and diagnostic accuracy for each category.

Materials and methods

This retrospective study, conducted from July 2023 to June 2024, analyzed respiratory cytology specimens, including bronchoalveolar lavage (BAL), bronchial washings (BW), endobronchial ultrasound-guided transbronchial needle aspiration (EBUS-TBNA), and fine-needle aspiration cytology (FNAC). Pleural effusions were excluded. Cytologic diagnoses were correlated with histopathology to determine ROM for individual categories.

Results

A total of 150 respiratory samples were analyzed, comprising BAL/BW (128), EBUS-TBNA (21), and ultrasonography* *(USG)-guided FNAC (one). Histopathologic correlation was available for 60 cases (40%). Based on the WHO classification, 28 cases (18.7%) were categorized as insufficient/inadequate/non-diagnostic, 109 (72.7%) as benign, four (2.7%) as suspicious for malignancy, and eight (5.3%) as malignant, and one (0.6%) was categorized as atypical. The estimated ROM for each category was 11.1% (non-diagnostic), 32.5% (benign), 66.6% (suspicious for malignancy), and 75% (malignant).

The specificity of BAL/BW and EBUS-TBNA was 100%. Sensitivity varied across sample types, with BAL/BW at 31.25% and EBUS-TBNA at 50%, resulting in an overall sensitivity of 36.4% and a diagnostic accuracy of 71.67%.

Conclusion

The WHO Reporting System for Lung Cytopathology standardizes diagnostic categorization, improving communication, facilitating risk stratification, and enhancing clinical decision-making. Its implementation supports more accurate malignancy risk assessment, improving patient care.

## Introduction

Lung cancer is the leading cause of cancer-related deaths and the second most commonly diagnosed cancer in both men and women [1]. Lung cytopathology plays a crucial role in detecting suspected malignancies, providing the best opportunity for early and effective treatment. Various cytological specimens, such as sputum, bronchial brushing (BB), bronchial washings (BW), bronchoalveolar lavage (BAL), and endobronchial ultrasound/computed tomography (EBUS/CT)-guided fine-needle aspiration cytology (FNAC), are essential for diagnosis. Standardized terminology and reporting systems ensure clear and consistent communication between cytopathologists and clinicians [2,3].

In 2016, the Papanicolaou Society of Cytopathology (PSC) introduced a six-tiered classification system for pulmonary cytology specimens, assigning a corresponding risk of malignancy (ROM) to each category [4,5]. In 2020, the Japan Lung Cancer Society and the Japanese Society of Clinical Cytology developed a four-tiered reporting system for lung carcinoma, which is widely used in Japan [6]. Despite these advancements, a universally accepted global standard has yet to be established.

To address this need, the International Academy of Cytology (IAC), in collaboration with the International Agency for Research on Cancer (IARC), published the World Health Organization (WHO) Reporting System for Lung Cytopathology in 2022 [7]. This system provides a standardized nomenclature and classification framework for lung cytology, offering precise definitions for each category, an associated ROM, and a recommended management algorithm [8]. Under this system, respiratory cytology specimens are classified into five diagnostic categories: insufficient/inadequate/non-diagnostic, benign, atypical, suspicious for malignancy, and malignant [9].

The "insufficient/inadequate/non-diagnostic" category refers to specimens with suboptimal cellularity or compromised morphology due to factors such as obscuring blood, inflammation, or inadequate fixation or staining. The "benign" category includes cytologic features indicative of non-neoplastic or benign neoplastic processes. The "atypical" category encompasses specimens with predominantly benign cytomorphology but with limited atypical features insufficient for a definitive diagnosis. "Suspicious for malignancy" denotes specimens with cytologic atypia suggestive of malignancy yet lacking sufficient criteria for a conclusive malignant diagnosis. The "malignant" category is reserved for specimens exhibiting unequivocal cytopathological features of malignancy [7].

This study aimed to analyze respiratory cytology specimens using the WHO Reporting System for Lung Cytopathology and evaluate the malignancy risk associated with each diagnostic category.

## Materials and methods

This was a retrospective observational study conducted at the Department of Pathology of NRI Medical College and Hospital, Guntur, India. The study analyzed archived cytology data collected routinely as part of the diagnostic work-up between July 2023 and June 2024. Although the samples and reports were generated during this period as part of routine diagnostic work, no data were analyzed or used for research until approval from the Institutional Ethics Committee (IEC) of NRI Medical College and General Hospital was obtained in 2025 (approval number: IEC 2025 F005). After obtaining ethical clearance, existing records were retrieved, and patient identifiers were anonymized and analyzed for research purposes. Clinical data, including patient age, sex, and histopathological diagnosis, were recorded.

Inclusion criteria

All respiratory cytology samples, including sputum, BAL, BW, BB, and guided FNAC procedures (EBUS/ultrasonography (USG)/CT), were included in the study.

Exclusion criteria

Pleural effusion samples and cases lacking adequate clinicopathological details were excluded from the study.

Sample processing

As per institutional protocols, a minimum of two smears were prepared from the sediment of centrifuged BAL and BW specimens and fixed in alcohol. Since the processing method and adequacy criteria for BAL and BW were similar, these modalities were grouped as BAL/BW in this study. In addition to smear preparation, samples were also collected for cell block preparation in tubes containing 10% neutral buffered formalin. All smears from EBUS-guided transbronchial needle aspiration (TBNA) and FNAC samples, along with alcohol-fixed smears, were stained using routine hematoxylin and eosin (H&E) staining and Papanicolaou staining.

Sample adequacy evaluation

BAL and BW were considered adequate if alveolar macrophages were readily identifiable. A BAL smear was deemed sufficient if it contained more than 10 alveolar macrophages per 2 mm² or 20 alveolar macrophages per 10 high-power fields [9,10]. Transthoracic FNAC samples were considered adequate if they contained alveolar macrophages (with or without hemosiderin), numerous nucleated bronchial epithelial cells, reactive type 2 pneumocytes, or tissue fragments from collapsed alveolar septa [9]. EBUS-guided transbronchial FNAC samples were deemed adequate if reactive bronchial ciliated columnar cells were present or if there were more than 40 lymphocytes per high-power field in the most cellular area [9].

Sample interpretation

Cytology samples were classified into one of the five diagnostic categories according to the WHO Reporting System for Lung Cytopathology. Patient demographics, clinical details, and diagnostic categories were documented and recorded as part of routine lab work. Of 150 cytology cases, 60 cases had corresponding biopsy specimens received in 10% neutral buffered formalin. The specimens were processed using routine lab protocol, sectioned, stained, and examined. 

Statistical analysis

Data were entered and tabulated using Microsoft Excel (Microsoft Corporation, Redmond, WA, USA) for statistical analysis. Descriptive statistics were applied to summarize the data. Categorical variables like sample type, cytological category, and histopathological diagnosis were expressed as percentages. Performance indicators, including sensitivity, specificity, positive predictive value (PPV), negative predictive value (NPV), and diagnostic accuracy (DA), were calculated using a standard 2×2 contingency table. Additionally, ROM for each WHO Reporting System for Lung Cytopathology diagnostic category was determined, considering histopathological diagnosis as the gold standard.

## Results

**Figure 1:**
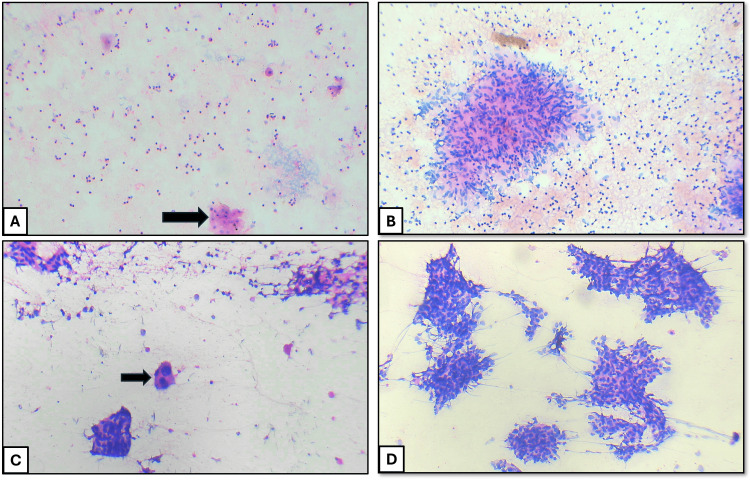
(A) Non-diagnostic: cytological smear showing only a few squamous epithelial cells (black arrow) and scattered inflammatory cells (H&E: 40×). (B) Benign: cytological smear showing an epithelioid granuloma and scattered lymphocytes (H&E: 100×). (C) Atypical: cytological smear showing benign bronchial epithelial cells with an atypical cell (black arrow) showing nucleomegaly and hyperchromatism (H&E: 100×). (D) Malignant: cytological smear showing sheets of atypical cells showing nuclear pleomorphism (H&E: 100×) H&E: hematoxylin and eosin

**Figure 2:**
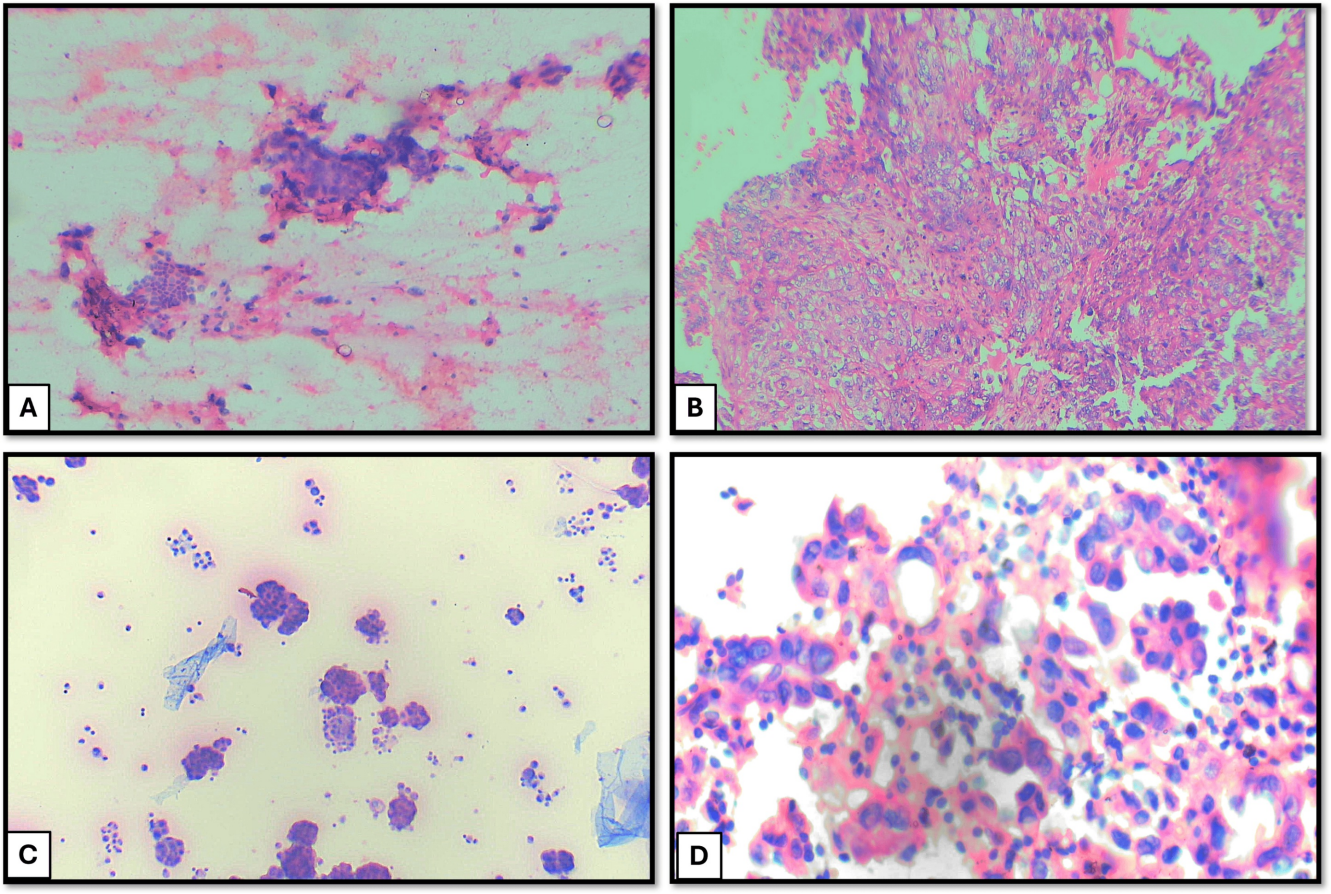
(A) Cytological smear showing atypical squamous cells in sheets and clusters suggestive of squamous cell carcinoma (H&E: 100×). (B) Cell block showing sheets of malignant squamous epithelial cells with pleomorphic nuclei and eosinophilic cytoplasm, suggestive of squamous cell carcinoma (H&E: 100×). (C) Cytological image showing malignant epithelial cells in glandular pattern and three-dimensional clusters suggestive of adenocarcinoma (H&E: 40×). (D) Cell block image showing malignant cells in glandular pattern suggestive of adenocarcinoma (H&E: 100×) H&E: hematoxylin and eosin

**Figure 3:**
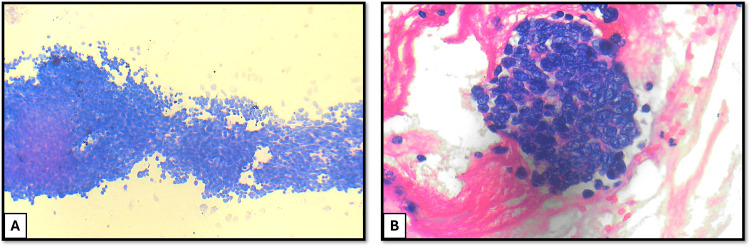
Cytology image of EBUS-guided FNAC showing monomorphic large atypical lymphoid cells with hyperchromatic nuclei and scant cytoplasm, suggestive of non-Hodgkin lymphoma (H&E: 40×). (B) Cell block image showing atypical lymphoid cells suggestive of non-Hodgkin lymphoma (H&E: 400×) EBUS: endobronchial ultrasound; FNAC: fine-needle aspiration cytology; H&E: hematoxylin and eosin

A total of 150 respiratory cytology samples were analyzed. Among these patients, 94 were males (62.7%), and 56 were females (37.3%). The majority of patients were in their sixth decade of life, with ages ranging from 14 to 83 years. Among the collected cytology samples, BAL and BW accounted for the majority (128/150, 85.3%), followed by EBUS-guided aspirations (21/150, 14%) and a single USG-guided FNAC sample (Table 1). All cases were classified according to the WHO Reporting System for Lung Cytopathology. The distribution of cases was as follows: 28 cases (18.7%) were categorized as non-diagnostic, 109 cases (72.7%) were categorized as benign, one case (0.6%) was categorized as atypical, four cases (2.7%) were categorized as suspicious for malignancy, and eight cases (5.3%) were categorized as malignant (Table 1). The cytological smear images for the individual category are shown in Figure 1. The cell block and cytological smear correlation images of malignant lesions are shown in Figure 2 and Figure 3.

**Table 1:** Number of cases in each diagnostic category across different types of samples BAL: bronchoalveolar lavage; BW: bronchial washings; EBUS-TBNA: endobronchial ultrasound-guided transbronchial needle aspiration; FNAC: fine-needle aspiration cytology

Diagnostic category	BAL/BW	EBUS-TBNA	FNAC	Total
Non-diagnostic/inadequate	22	5	1	28 (18.7%)
Benign	99	10	-	109 (72.7%)
Atypical	1	-	-	1 (0.6%)
Suspicious for malignancy	3	1	-	4 (2.7%)
Malignant	3	5	-	8 (5.3%)
Total cases	128	21	1	150

Table 2 summarizes the histopathological correlation and corresponding ROM across various cytology diagnostic categories classified according to the WHO Reporting System for Lung Cytopathology. Histopathological follow-up was available for 60 cases. Among the non-diagnostic category (n=9), five (55.6%) were inadequate on histopathology, three (33.3%) were benign, and one (11.1%) was malignant, yielding a ROM of 11.1%. In the benign cytology category (n=40), histopathology showed three (7.5%) inadequate, 24 (60%) benign, and 13 (32.5%) malignant outcomes, with a ROM of 32.5%. No follow-up biopsy was available for the atypical category (n=0); hence, ROM could not be calculated. Of the suspicious for malignancy cases (n=3), histopathology confirmed malignancy in two (66.7%), while one was inadequate, giving a ROM of 66.7%. Among malignant cytology cases (n=8), histopathology confirmed malignancy in six (75%), while two (25%) were inadequate resulting in a ROM of 75%. Overall, the ROM showed a progressive increase from the non-diagnostic to the malignant category, reflecting the predictive reliability of the WHO Reporting System for Lung Cytopathology in stratifying malignancy risk.

**Table 2:** Histological correlation and ROM among different WHO lung cytology categories HPE: histopathological examination; ROM: risk of malignancy; WHO: World Health Organization

Cytology diagnostic category	HPE opinion-inadequate	HPE opinion-benign	HPE opinion-malignant	ROM (%)
Non-diagnostic (n=9)	5 (55.6%)	3 (33.3%)	1 (11.1%)	11.1%
Benign (n=40)	3 (7.5%)	24 (60%)	13 (32.5%)	32.5%
Atypical (n=0)	Nil	Nil	Nil	Not calculated
Suspicious for malignancy (n=3)	1 (33.3%)	0 (0%)	2 (66.7%)	66.7%
Malignant (n=8)	2 (25%)	0 (0%)	6 (75%)	75%

Sensitivity, specificity, PPV, NPV, and DA for all samples, as well as for BAL/BW and EBUS-TBNA samples, are summarized in Table 3. The overall specificity and PPV were 100%, indicating a high reliability in identifying true positives when malignancy was detected on cytology. However, the sensitivity was relatively low at 36.36% with the lowest (31.25%) seen in BAL/BW specimens, reflecting a high rate of false negatives in these samples. EBUS-FNAC demonstrated a better sensitivity (50%) compared to BAL/BW showing superior diagnostic yield in detecting malignancy. However, the overall DA was 72%, suggesting that cytology has high specificity, but its ability to rule out malignancy is limited. These findings emphasize the need for comprehensive clinical and radiological correlation, especially in cytologically benign cases where there is a high index of clinical suspicion of malignancy.

**Table 3:** Diagnostic performance among various types of samples and overall respiratory cytology samples PPV: positive predictive value; NPV: negative predictive value; DA: diagnostic accuracy; BAL: bronchoalveolar lavage; BW: bronchial washings; EBUS-TBNA: endobronchial ultrasound-guided transbronchial needle aspiration

Diagnostic performance	Sensitivity	Specificity	PPV	NPV	DA
Overall	36.36%	100%	100%	66.6%	72%
BAL/BW	31.25%	100%	100%	69.4%	73.2%
EBUS-TBNA	50%	100%	100%	50%	66.6%

## Discussion

Respiratory cytology serves as a critical diagnostic tool for lung cancer, since approximately 70% of cases are inoperable at the time of diagnosis [11,12]. Given this limitation, accurate detection of primary lung cancer through biopsy and cytology is essential for guiding appropriate patient management. The WHO Reporting System for Lung Cytopathology standardizes terminology to ensure clear communication between cytopathologists and clinicians. It helps assess sample adequacy, guide further tests, and support accurate diagnosis for better patient care.

This study aimed to assess the ROM across different categories of lung cytology. Table 4 presents a comparison of the ROM results from the current study with those from other published studies. In our study, the ROM for the non-diagnostic category was 11.1%, which was significantly lower than the rates reported in previous studies by O'Connor et al. (37.7%) [12], Layfield et al. (40%) [4], Canberk et al. (64.01%) [8], and Meena et al. (49.2%) [13]. The WHO-estimated ROM for this category, based on published data, ranges from 40% to 60% [7]. The ROM for the benign category was 32.5%, which aligns with findings from Layfield et al. (20%) [4] and Canberk et al. (40%) [8] but is higher than the rates reported by Hiroshima et al. (19.3%) [6], O'Connor et al. (18.1%) [12], Meena et al. (13.3%) [13], and Yoshizawa et al. (17.7%) [14]. The ROM for the atypical category could not be determined, as no biopsy correlation or follow-up was available for the single case identified. The ROM for the suspicious for malignancy and malignant categories was 66.6% and 75%, respectively, which were lower than the findings reported by Meena et al. (81.5% and 92.7%, respectively) [13] and the data given in the WHO Reporting System for Lung Cytopathology [7]. Furthermore, the proportion of cases in the suspicious for malignancy and malignancy categories was relatively low (2.6% and 5.3%, respectively) in our study compared to WHO data, which estimates these categories at 5% and 20%, respectively [7]. Consistent with findings from previous studies, our data demonstrated a progressive increase in ROM from the benign to the malignant category [5,13,15]. This reinforces the validity of cytologic categorization in estimating ROM, thereby aiding in risk stratification and facilitating timely clinical decisions.

**Table 4:** Comparison of overall ROM for each category among various studies WHO: World Health Organization; ROM: risk of malignancy

Diagnostic category	Present study	Meena et al. [13]	O'Connor et al. [12]	Layfield et al. [4]	Canberk et al. [8]	WHO [7]
Inadequate/non-diagnostic	11.1%	49.2%	37.7%	40%	64.01%	40-60%
Benign	32.5%	13.3%	18.1%	20%	48.3%	20-40%
Suspicious for malignancy	66.6%	81.5%	85.7%	82%	90%	82%
Malignant	75%	92.7%	91.9%	77-100%	89.74%	90%

In this study, sensitivity, specificity, PPV, NPV, and DA were calculated for different cytology modalities. Atypical, suspicious for malignancy, and malignant cases were considered positive. Both BAL/BW and EBUS-TBNA samples demonstrated 100% specificity, which is consistent with the findings of O'Connor et al. [12] and Meena et al. [13], who also reported 100% specificity for all modalities. The overall sensitivity in our study was 36.36%, with BAL/BW showing a sensitivity of 31.25%, which is comparable to the 18.9% reported by O'Connor et al. [12]. The sensitivity of EBUS-TBNA was 50%, which was lower than the 100% sensitivity reported by O'Connor et al. [12] and Meena et al. [13] for EBUS-TBNA.

Given the low sensitivity of BAL/BW samples, these should not be used as a standalone diagnostic tool for excluding malignancy. Instead, a comprehensive evaluation that incorporates clinical, radiological, and microbiological correlations is essential. The high PPV of respiratory cytology samples reinforces the reliability of the material obtained. Adhering to WHO adequacy guidelines and diagnostic categories has improved communication with clinicians regarding sample adequacy and the need for repeat sampling. This has resulted in shorter procedure times, fewer needle passes, and a reduction in patient complications. Given the scarcity of studies utilizing the WHO Reporting System for Lung Cytopathology, our study offers valuable insights into ROM and performance indicators. However, multicentric studies are required to further validate and expand upon these findings.

Limitations

The primary limitations of this study include a limited sample size and restricted histopathological correlation with biopsy confirmation available in 40% of cases. Only one case was categorized as atypical, and follow-up biopsy data for this case was not available. 

## Conclusions

The implementation of the WHO Reporting System for Lung Cytopathology in our study proved effective in standardizing terminology and risk assessment, ultimately enhancing clinical decision-making and patient care. Among 150 samples, we categorized 28 (18.7%) as non-diagnostic, 109 (72.7%) as benign, one (0.6%) as atypical, four (2.7%) as suspicious for malignancy, and eight (5.3%) as malignant. Histopathological correlation was available for 60 cases, demonstrating 100% specificity for both BAL/BW and EBUS-TBNA specimens, with EBUS-TBNA showing higher sensitivity than BAL/BW samples. The calculated ROM was 11.1% for non-diagnostic cases, 32.5% for benign cases, 66.6% for suspicious for malignancy cases, and 75% for malignant cases. This progressive increase in ROM underscores the value of cytologic classification in risk stratification.
